# Can community retail pharmacist and diabetes expert support facilitate insulin initiation by family physicians? Results of the AIM@GP randomized controlled trial

**DOI:** 10.1186/1472-6963-13-71

**Published:** 2013-02-21

**Authors:** Stewart B Harris, Hertzel C Gerstein, Jean-François Yale, Lori Berard, John Stewart, Susan Webster-Bogaert, Jordan W Tompkins

**Affiliations:** 1 Centre for Studies in Family Medicine, Department of Family Medicine, Schulich School of Medicine & Dentistry, The University of Western Ontario, 245-100 Collip Circle, London, Ontario, N6G 4X8, Canada; 2 Department of Medicine, McMaster University, Health Sciences Centre Room 3 V38, 1280 Main Street West, Hamilton, Ontario, L8S 4K1, Canada; 3 Department of Medicine, McGill University, Royal Victoria Hospital, 687 Pine Avenue West, M9.05, Montreal, Quebec, H3A 1A1, Canada; 4 Health Sciences Centre, 820 Sherbrook Street, Winnipeg, Manitoba, R3A 1R9, Canada; 5 sanofi-aventis, 2150 St. Elzear Blvd. West, Laval, Quebec, H7L 4A8, Canada

**Keywords:** Clinical inertia, Family practice, Insulin, Pharmacists, Type 2 diabetes mellitus

## Abstract

**Background:**

Limited evidence exists on the effectiveness of external diabetes support provided by diabetes specialists and community retail pharmacists to facilitate insulin-prescribing in family practice.

**Methods:**

A stratified, parallel group, randomized control study was conducted in 15 sites across Canada. Family physicians received insulin initiation/titration education, a physician-specific ‘report card’ on the characteristics of their type 2 diabetes (T2DM) population, and a registry of insulin-eligible patients at a workshop. Intervention physicians in addition received: (1) diabetes specialist/educator consultation support (active diabetes specialist/educator consultation support for 2 months [the educator initiated contact every 2 weeks] and passive consultation support for 10 months [family physician initiated as needed]); and (2) community retail pharmacist support (option to refer patients to the pharmacist(s) for a 1-hour insulin-initiation session). The primary outcome was the insulin prescribing rate (IPR) per physician defined as the number of insulin starts of insulin-eligible patients during the 12-month strategy.

**Results:**

Consenting, eligible physicians (n = 151) participated with 15 specialist sites and 107 community pharmacists providing the intervention. Most physicians were male (74%), and had an average of 81 patients with T2DM. Few (9%) routinely initiated patients on insulin. Physicians were randomly allocated to usual care (n = 78) or the intervention (n = 73). Intervention physicians had a mean (SE) IPR of 2.28 (0.27) compared to 2.29 (0.25) for control physicians, with an estimated adjusted RR (95% CI) of 0.99 (0.80 to 1.24), *p* = 0.96.

**Conclusions:**

An insulin support program utilizing diabetes experts and community retail pharmacists to enhance insulin prescribing in family practice was not successful. Too few physicians are appropriately intensifying diabetes management through insulin initiation, and aggressive therapeutic treatment is lacking.

**Trial registration:**

ClinicalTrial.gov: NCT00593489

## Background

The majority of patients with type 2 diabetes mellitus (T2DM) are managed in the family practice setting [1], but approximately 50% of these patients do not meet guideline-recommended glycemic targets [2,3]. Clinical practice guidelines emphasize insulin therapy for T2DM as an appropriate therapy at any point when glycemic targets are not met [4,5]. Recent studies of primary care physicians in Canada have found that only 12% [2] to 15% [3] of patients with T2DM were prescribed insulin (with or without oral agents) while the literature suggests suggest that up to 60% of patients may eventually require insulin therapy [6]. These data indicate that family physicians remain uncomfortable initiating and managing insulin for their patients. Indeed, in a survey on patient and healthcare provider attitudes towards insulin, most nurses and general practitioners (50-55%) reported that they delay insulin treatment until absolutely necessary [7].

Physician, patient, and contextual factors interact to influence physician prescribing behaviors [8-11]. Physician -level barriers include: lack of knowledge and clinical skills for diabetes management [8]; time constraints [12,13]; ineffective charting systems [13-15]; clinical inertia [16,17]; and absence of organizational systems that allow adequate time and resources. In addition, physicians resist prescription of insulin due to concerns about patient weight gain, hypoglycemia, and patient expectations and fears, including injection anxiety.

The delivery of primary healthcare services is in transition in Canada. A key feature of this reform is the inclusion of pharmacists and other interdisciplinary allied health professionals within family healthcare teams [18-21]. The pharmacy profession is also experiencing rapid change in the Canadian healthcare system. Transition in scope of practise to include limited prescribing rights has occurred or has been proposed in a number of Canadian provinces [22-24] following other international jurisdictions [25]. Furthermore, in Canada, pharmacists constitute the fastest growing group of certified diabetes educators [26]. Direct integration of pharmacists into a family practice setting has been shown to positively affect physician-pharmacist collaboration and patient outcomes [27-30], however the effectiveness of support provided by community retail pharmacists on patient medical outcomes is inconclusive [31-33].

The objective of the Advancing Insulin Management in General Practice (AIM@GP) trial was to determine the effectiveness of an insulin initiation strategy utilizing diabetes specialist and community retail pharmacy support to increase family physician insulin prescribing rates. The strategy targeted recognized major barriers to physician insulin prescribing: time constraints; system support; and clinical inertia. Providing access to a diabetes expert had the potential to act as a reminder system and maintain knowledge gained from a workshop. Providing access to a community retail pharmacist had the potential to be time-saving and an alternative organizational system for the insulin initiation process.

## Methods

A stratified, parallel group, randomized controlled study was conducted in family physician clinics and community pharmacies across Canada from July 2006 to April 2010. The University of Western Ontario Centre for Studies in Family Medicine served as the Coordinating Centre. Ethics approval was obtained from academic ethics committees across Canada including The University of Western Ontario.

Family physician enrollment occurred from July 2006 to September 2008. Physicians invited to participate were randomly selected from a published national directory [34]. Physicians provided written, informed consent, and were screened for eligibility and included: full time status [>25 hours/week]; ability to generate a list of patients with T2DM [ICD-9, 250 billing code]; minimum of 35 patients with T2DM, 4 insulin-eligible patients in the practice; and attendance at a mandatory workshop. Physician study exclusion criteria included: academic practice; participation in a diabetes behaviour-change intervention trial; planned retirement or moving practice to another city; or planned extended locum coverage beyond 4 weeks during the subsequent 12 month intervention period. Enrollment ended when all physicians assessed for eligibility consented, refused consent, or were deemed unable to follow-up (i.e. moved).

Community pharmacist recruitment was conducted utilizing a national registry at the Department of Family Medicine at McMaster University. Potential pharmacists in geographic proximity to the postal codes of consenting physicians were identified and surveyed to determine their diabetes education training and pharmacy services and resources.

Physicians generated a list of all patients with T2DM in their practice and mailed study information to all. Study auditors reviewed the charts of patients who provided written informed consent and collected year of birth, year of T2DM diagnosis, glycosylated haemoglobin (HbA1c) values and glucose-lowering medications. The remaining charts of patients who either, did not consent or did not respond, were reviewed by the physician to determine insulin-eligibility. Patients were considered insulin-eligible if they had an HbA1c ≥7.5% (most recent laboratory value) and their oral anti-diabetes drug (OAD) score was ≥1.5 (OAD score is the sum of all OADs prescribed; OAD ½ to maximum dose = 1 OAD) [35]. Lantus® or NPH insulin was available free of charge for 6 months to all insulin-eligible patients.

All eligible physicians were stratified by the study geographic site and their level of comfort prescribing insulin (determined by questionnaire) and randomly allocated (1:1) in a blocked manner to an insulin initiation strategy (intervention) or usual care (control) by the Coordinating Center. Sanofi-aventis generated the mechanism used to implement the random allocation sequence. A follow-up chart audit commenced 15 months post workshop (defined for each physician; January 2007 – March 2010).

Study sites hosted insulin initiation workshops for all physicians enrolled in the study to ensure comparable knowledge on the appropriate use of insulin therapy in T2DM. All physicians received a complete registry of insulin-eligible patients in their practice. Pharmacists attended the same program but were educated separately to avoid contamination. The workshop focussed on education regarding glycemic control, the rationale for insulin prescription, and common patient barriers. Additional activities included viewing of an insulin initiation video and hands-on experience with an insulin pen. For physicians, summary chart audit data were presented and individual practice-specific ‘report cards’ distributed. Physicians were notified of their randomization status at the workshop by the Coordinating Center. Intervention physicians had the opportunity to meet pharmacist(s) with whom they were matched.

### Intervention – insulin initiation strategy

Physicians randomized to the intervention group were provided with a 12-month insulin initiation strategy consisting of (1) diabetes specialist/educator consultation support (active diabetes specialist/educator consultation support for 2 months [the educator initiated contact every 2 weeks] and passive consultation support for 10 months [family physician initiated as needed]); and (2) community retail pharmacist support (option to refer patients to the pharmacist(s) for a 1-hour insulin-initiation session). The session checklist included education on insulin action, injection sites, the pen device, and hypoglycemia awareness and treatment.

### Primary outcome

The primary outcome was the physician’s insulin prescribing rate (IPR)—the number of insulin starts per the 12-month intervention of insulin-eligible patients.

### Secondary outcomes

Secondary clinical outcome measures, limited to the data from the audit of consenting patients’ charts, included: HbA1c; fasting plasma glucose (FPG); OAD prescription and score; insulin prescription and dosage; proportion of patients at HbA1c target (≤7.0%); and proportion of patients with intensification of diabetes management (i.e. increased dose of OAD or insulin, increased OAD score, or the addition of insulin). For those patients prescribed insulin during the intervention, additional outcomes included: number of days from study start to insulin initiation; the change of HbA1c, FPG, and OAD score; and type(s) of insulin prescribed from initiation of insulin to 3 and 6 months post-initiation.

The physician knowledge, attitude and self-efficacy questionnaire was used to create change scores (pre – post intervention) to measure physician knowledge, attitude and self-efficacy [36,37] for both glycemia control and insulin initiation and titration. This questionnaire was developed by the Coordinating Centre for this project and tested for content validity (example questions are provided in Table 1).

**Table 1:** Example questions from the knowledge attitude and self-efficacy questionnaire

**Questionnaire section**	**Example questions / Statements**
**Attitude**	There is little point in trying to achieve optimal glucose because complications from diabetes are inevitable
5 point Likert Scale (Strongly Agree to Strongly Disagree)	Diabetes is harder to treat than other chronic diseases
	I will not prescribe insulin because I believe it is too complicated to initiate
**Self-Efficacy**	How confident are you to give adequate support and clear directions to patients on how to manage diabetes
5 point Likert Scale (Not at All to Completely)	How confident are you to prescribe and titrate insulin when a patient is anxious and is resists initiating insulin therapy
**Knowledge**	Diabetes related complications for both Type 1 and Type 2 could be prevented through optimal glucose control
Multiple Choice & True/False	Insulin replacement therapy with type 2 diabetes may be required for most patients during the duration of their disease.

### Sample size calculation

With a standard deviation of 7.1 estimated from Poisson regression, two-sided alpha of 0.05 and power of 0.90, the sample size required was 89 physicians per group (n = 178). Protocol modifications due to recruitment challenges included inclusion of group practice physicians (excluded in original protocol), and reduction of the minimum required number of patients with T2DM (original protocol = 50) and insulin-eligible patients (original protocol = 8).

### Analysis

The unit of analysis was the physician. Intention-To-Treat analysis was performed on the primary outcome with the IPR imputed as zero if data were not available. The analyses of secondary outcomes were based on all available data. A *p* value of ≤ 0.05 was deemed statistically significant. Analyses were generated using SAS Version 9.1. All data were examined and the appropriate analyses were employed.

#### Primary outcome

The IPR was analyzed using Poisson regression with intervention group as a class effect and mean HbA1c at baseline as a covariate from which the mean number of people started on insulin per 12 months, standard error (SE) and 95% confidence intervals (CIs) were calculated.

#### Secondary outcomes

Continuous variable changes from workshop to 15 months post-workshop were examined using analysis of covariance (ANCOVA) with the intervention group, baseline mean HbA1c, insulin comfort stratum and pooled site as class effects, and corresponding baseline value of the variable of interest as a covariate.

ANOVA procedures were used to compare intervention and control baseline physician and practice demographics for continuous variables and chi-square tests for categorical variables. Knowledge, attitude and self-efficacy change scores were analyzed using one-way ANOVA.

## Results and discussion

### Results

**Figure 1:**
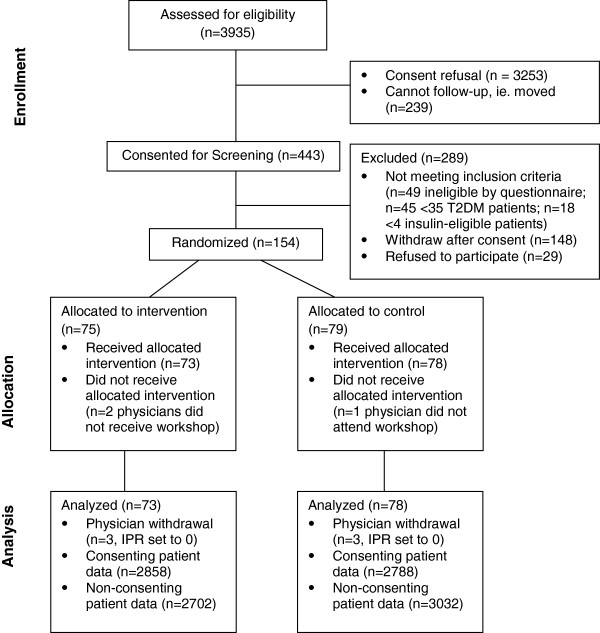
CONSORT diagram - disposition of physician subjects.

Of 443 family physicians who consented for screening, 154 were randomized and 151 were included in the final analysis (n = 73 in the intervention group, n = 78 in the control group). Three physicians withdrew prior to attending the workshop, all were blind to which group they were allocated. An additional 6 physicians withdrew after attending the workshop, prior to the final chart audit. Data were collected from 11,380 patient charts. See Figure 1 for the disposition of physician subjects and patient consent distribution. Fifteen specialist sites and 107 community retail pharmacists were available to the intervention group physicians.

Table 2 outlines baseline demographics of physicians and practice characteristics by study group. Physicians were comparable in their demographics with the exception that intervention physicians were older (*p* = 0.03), reported seeing significantly more patients per day (*p* = 0.02), had been in practice longer (*p* = 0.05), and reported a lower level of diabetes-related Continuing Medical Education attendance (*p* < 0.01) than control physicians. IPR subgroup analyses to account for differences in baseline demographics showed no significant impact on the primary outcome and therefore these were not controlled for in any subsequent analyses.

**Table 2:** Baseline demographic characteristics of physicians (n = 151)

**Physicians**	**Intervention**	**Control**	*p *value*
	**n = 73**	**n = 78**	
Mean age in years (SD)	51.4 (8.81)	48.2 (9.06)	0.03
Male (n,%)	53 (72.6)	59 (75.6)	0.67
Urban (n,%)	56 (76.7)	61 (78.2)	0.83
Mean years in practice (SD)	26.1 (8.79)	23.1 (9.55)	0.05
No DM CME^**^ Attendance (n,%)	21 (38.2)	7 (11.1)	< 0.01
Mean number of patients per physician (SD)	2440 (1239)	2365 (1206)	0.72
Mean number of patients seen per day per physician (SD)	39.6 (13.15)	35.0 (8.95)	0.02
Mean number of patients with T2DM per physician (SD)	82.1 (38.57)	80.6 (36.30)	0.81
Did not routinely initiate insulin (n,%)	68 (93.2)	69 (88.5)	0.32

*One way ANOVA or chi-square test. **CME – diabetes related continuing medical education.

Patient summary data (Table 3) were computed for consenting patients (49.6% consent rate) and were comparable between study groups. Overall glycemic control, as measured by mean HbA1c, was excellent in both study groups, 7.11% and 7.19% for consenting patients in the intervention and control groups, respectively.

**Table 3:** Patient summary statistics

	**Intervention**	**Control**	***p *value**
All Patients* (n)	5560	5820	
Consenting Patients** (n)	2858	2788	
Mean HbA1c at baseline (SD)**	7.11 (0.43)	7.19 (0.52)	0.32
Mean years since T2DM diagnosis (SD)**	7.9 (2.54)	8.0 (2.90)	0.80
Mean number of insulin-eligible patients at time of workshop per physician (n,%)	10.9 (5.80)	11.3 (7.09)	0.70
Mean HbA1c of insulin-eligible patients at time of workshop (SD)**	8.6 (0.59)	8.6 (0.76)	0.74
Mean number of patients newly prescribed insulin between Workshop and 12 months post (SD)**	1.8 (1.87)	1.9 (1.71)	0.91
Mean number of office visits during study for insulin-eligible patients at the time of insulin initiation (SD)	6.0 (2.24)	5.9 (2.39)	0.84
Mean elapsed time in days, workshop to first office visit for insulin-eligible patients at the time of insulin initiation (SD)	45.3 (32.92)	65.4 (53.99)	0.04

*All patients with T2DM in practice.

**Data from consenting patients’ charts only.

#### Primary outcome

Intervention physicians had a mean (SE) IPR of 2.28 (0.27) compared with 2.29 (0.25) for the control physicians, with an estimated adjusted RR (95% CI) of 0.99 (0.80 to 1.24), *p* = 0.96. No significant differences were found between the two groups.

#### Secondary outcomes

Statistically significant within-group differences for both intervention and control physicians revealed a higher percentage of patients prescribed OADs, OAD score, and increased percentage of patients prescribed insulin at 15 months (*p* < 0.05). Intervention physicians significantly increased the insulin daily dose (*p* < 0.01) by15 months (Table 4). Secondary analyses of between-group differences of consenting patient charts showed an increase at 15 months in the mean daily dose of insulin prescribed by intervention physicians, with an adjusted mean (SE) of 5.96 (2.83), 95% CI (0.35 to 11.56), *p* = 0.04. No other treatment effects between intervention and control groups were evident (Table 4).

**Table 4:** Within and between-group change from workshop to end of study (15 months)

	**Group**	**Descriptive statistics Mean (SD)**		**Change at 15 months: *Within-group p*-value**	**Change at 15 months: Between groups**		
		**Workshop**	**Final**		**Adjusted mean (SE)**	**95% CI**	***Between-group p*-value**
HbA1c^*^,%	I (n = 73)	7.12 (0.42)	7.09 (0.41)	0.34	0.02 (0.04)	(−0.05 to 0.09)	0.63
	C (n = 76)	7.20 (0.52)	7.15 (0.51)	0.13			
% patients HbA1c ≤ 7.0%	I (n = 73)	58.40 (16.20)	58.68 (15.40)	0.76	−1.22 (1.30)	(−3.78 to 1.35)	0.35
	C (n = 76)	55.95 (15.65)	57.58 (16.12)	0.15			
FPG^†^ level in mmol/L	I (n = 71)	7.86 (0.67)	7.78 (0.68)	0.13	0.01 (0.07)	(−0.13 to 0.15)	0.85
	C (n = 70)	7.89 (0.69)	7.82 (0.68)	0.22			
% patients prescribed OAD^‡^	I (n = 73)	80.87 (10.83)	83.32 (9.98)	< 0.001	0.87 (0.65)	(−0.42 to 2.16)	0.18
	C (n = 76)	79.24 (12.06)	81.12 (11.14)	< 0.001			
OAD score^§^	I (n = 73)	1.32 (0.20)	1.37 (0.21)	< 0.001	0.01 (0.02)	(−0.03 to 0.04)	0.65
	C (n = 76)	1.38 (0.21)	1.41 (0.24)	0.02			
% patients prescribed insulin	I (n = 73)	11.53 (8.93)	17.14 (10.64)	< 0.001	−1.06 (0.88)	(−2.80 to 0.69)	0.23
	C (n = 76)	11.53 (8.51)	18.38 (10.82)	< 0.001			
Insulin daily dose (units)	I (n = 61)	47.91 (21.24)	53.64 (22.71)	< 0.01	5.96 (2.83)	(0.35 to 11.56)	0.04
	C (n = 57)	57.19 (26.21)	54.94 (23.65)	0.30			
Intensification of diabetes management^**^	I (n = 73)	--	31.59 (14.17)	--	−0.29 (2.35)	(−4.93 to 4.34)	0.90
	C (n = 76)	--	32.80 (14.91)	--			

Note: CI = confidence interval, ANCOVA = analysis of covariance with treatment, stratum, and pooled site effects and the corresponding baseline value as covariate used for the change from baseline (difference: endpoint-baseline) with *p*-values based on actual data.

^*^HbA1c – glycosylated hemoglobin.

^†^FPG – fasting plasma glucose.

^‡^OAD – oral anti-diabetes drug.

^§^OAD score = sum of all OADs prescribed; OAD ½ to maximum dose = 1 OAD.

^¶^These are *between-group* results where change from workshop to 15 months was compared between intervention and control groups using ANCOVA, treatment effect is adjusted for baseline effect and pooled site effect.

^**^Intensification of diabetes management – Increased dose of OAD or insulin, increased OAD score, or the addition of insulin; Intensification is a one-time post workshop variable, therefore no within group differences.

Analyses of patients insulin-eligible at the time of insulin initiation found no significant treatment effects between intervention and control group physicians at 3 or 6 months post insulin initiation; however within-group reductions (*p* < 0.05) were found across both groups for HbA1c, FPG and OAD score at 3 and 6 months post initiation. The elapsed number of days between the workshop to first office visit for patients insulin-eligible at the time of insulin initiation was significantly lower for intervention physicians (45.3 days, SD = 32.92), compared with control physicians (65.4 days, SD = 53.99), *p* = 0.04 (Table 3).

At 15 months, the change in physician’s knowledge, attitude and self-efficacy of glycemia control and insulin initiation and titration did not differ between study groups. Statistically significant within-group differences at 15 months showed increased knowledge, attitude and self-efficacy of insulin initiation and titration for both groups. Intervention and control physicians also displayed increased self-efficacy of glycemic control, *p* < 0.01. Increased knowledge of glycemia control was limited to intervention physicians, *p* < 0.01.

### Discussion

Offering family physicians the option to utilize community retail pharmacists for insulin initiation with back-up support by a specialist team did not result in a significant improvement in insulin prescribing behaviour. This trial, therefore, provides no evidence to support a change in strategy for initiating insulin therapy in T2DM in family practice by providing an external expert support structure.

These findings are supported by previous literature showing lack of improvement in patient outcomes by interventions involving community retail pharmacists [32,33,38]. Pharmacists are highly qualified professionals with a strong interest in diabetes care. Thus there is a need to develop and evaluate how to optimize their inclusion in collaborative diabetes care. A better use of healthcare resources with improved clinical outcomes could come from supporting the direct integration of pharmacists into the family practice setting, an organisational structure supported in the literature [30,39,40].

There are several possible factors that may have contributed to the lack of effect of the intervention. The number of insulin-eligible patients for both intervention and control groups was lower than anticipated, limiting the total number of patients for physicians to act upon. There may also have been minimal pressure for physicians to act on the insulin-eligible patients who were only moderately out of target (i.e. with an HbA1c ≥7.5%). Clinical practice guidelines recommend aggressive treatment of patients with an HbA1c >7.0% [4,5], however a recent study examining clinical inertia in patients with T2DM revealed a high mean HbA1c of 9.5% at the time of insulin initiation [41]. Regardless of the sufficiently high risk HbA1c, physicians often pursue other glycemia-lowering options before turning to insulin [7]. A recent Canadian study of family physicians in practice for less than ten years demonstrated a greater willingness to share clinical information and communicate with pharmacists compared to more established family physicians [42]. The physicians in this study were older and had been in practice a mean duration of 26 and 23 years and thus may have had more difficulty adopting the intervention. The significant improvement of physicians’ knowledge, attitude and self-efficacy of insulin initiation and titration demonstrates the strength of the workshop for both groups and may potentially have impacted on the differences between groups. Clinical inertia, the lack of clinical action when one has the knowledge and opportunity [16], was however still evident in both groups with a lower than expected rate of insulin initiation in patients identified as being insulin-eligible in their practices.

#### Limitations

Participation bias may have led to the inclusion of physicians with a more active interest in insulin initiation. However the randomized controlled design ensured that participation bias occurred in both groups. The estimated sample size of 89 physicians per group was not achieved hence the final results may have been underpowered. However, as the means were exactly the same, it was unlikely that even with sufficient power that a difference would have been detected. Risk of contamination of the control physicians could have occurred if they practiced in the same location as an intervention physician. However, this situation occurred for only 3 control physicians thus we do not believe it affected the overall results. In addition, diabetes specialists and pharmacists did not provide support to the control physicians in the study. The “Hawthorne” effect, which suggests that physicians in a research study may be influenced by the belief that they are being monitored and change their behaviour accordingly [43] may have also had an effect, thus making it difficult to detect a statistically significant difference. Lastly, pharmacist recruitment challenges delayed the start of the intervention for some physicians, perhaps impacting the intervention and potential outcomes.

## Conclusion

The approach of pairing family physicians with diabetes experts and community retail pharmacists as a facilitating infrastructure to enhance insulin prescribing behaviour in family practice was not successful. Too few family physicians are appropriately intensifying glycemia management through insulin initiation, and aggressive therapeutic treatment is lacking. Primary care reform models promoting the interdisciplinary support of healthcare teams for chronic disease management including integrating pharmacists into the family practice setting, a strategy that has been shown to positively affect outcomes, may constitute a better use of healthcare resources.

## Abbreviations

AIM@GP: Advancing Insulin Management in General Practice; T2DM: Type 2 diabetes; IPR: Insulin prescribing rate; HbA1c: Glycosylated haemoglobin; FPG: Fasting plasma glucose; OAD: Oral anti-diabetes drug; CI: Confidence interval; ANCOVA: Analysis of covariance; SE: Standard error; SD: Standard deviation.

## Competing interests

Dr. Harris has received honoraria for consulting and speaking from sanofi-aventis, and his institution has received research funding for this project. Dr. Gerstein has received honoraria for consulting and speaking from sanofi-aventis and his institution has received research funding for a trial that he is leading. Dr. Yale has received honoraria for conferences and advisory boards from sanofi-aventis. Lori Berard received consulting fees related to this project. John Stewart is an employee of sanofi-aventis. Susan Webster-Bogaert and Jordan Tompkins are employed by Dr. Harris’ institution and received salary. The authors declare no other financial relationships with any organisations that might have an interest in the submitted work, and no other relationships or activities that could appear to have influence the submitted work.

## Authors’ contributions

SH was the lead investigator, oversaw the conception and design of the study, assisted with collection and interpretation of data, and was responsible for advising manuscript writers and editors during the creation and revision of the paper. HG, JFY and LB assisted with study design and interpretation of data. JS provided input in the study design and was responsible for analyzing the data and assisting with data interpretation. SWB was the project research coordinator and was involved in the study design, project management including data collection, and assisted in the interpretation of data and managing the manuscript revisions. JT assisted with the study design, project management including database supervision and data collection, assisted in the interpretation of data, and was involved in manuscript preparation, revision and submission. All of the authors approved the final version submitted for publication and acted independently from the study sponsor. All authors read and approved the final manuscript.

## Pre-publication history

The pre-publication history for this paper can be accessed here:

http://www.biomedcentral.com/1472-6963/13/71/prepub
